# Rapid Isolation and Determination of Flavones in Biological Samples Using Zinc Complexation Coupled with High-Performance Liquid Chromatography

**DOI:** 10.3390/molecules21081067

**Published:** 2016-08-16

**Authors:** Chenghe Sun, Hecheng Wang, Yingping Wang, Shengyuan Xiao

**Affiliations:** 1 Institute of Special Wild Animals and Plants, Chinese Academy of Agricultural Science, Changchun 130122, China; 18611297241@126.com (C.S.); whcxf332@163.com (H.W.); yingpingw@126.com (Y.W.); 2 School of Life Science, Beijing Institute of Technology, Beijing 100081, China

**Keywords:** *Scutellaria baicalensis*, dried blood spot, metal complexation, high-performance liquid chromatography, chlorophyll, heme

## Abstract

Chlorophyll-type contaminants are commonly encountered in the isolation and determination of flavones of plant aerial plant parts. Heme is also a difficult background substance in whole blood analysis. Both chlorophyll and heme are porphyrin type compounds. In this study, a rapid method for isolating flavones with 5-hydroxyl or *ortho*-hydroxyl groups from biological samples was developed based on the different solubilities of porphyrin-metal and flavone-metal complexes. It is important that other background substances, e.g., proteins and lipids, are also removed from flavones without an additional processing. The recoveries of scutellarin, baicalin, baicalein, wogonoside and wogonin, which are the primary constituents of *Scutellaria baicalensis* (skullcaps) were 99.65% ± 1.02%, 98.98% ± 0.73%, 99.65% ± 0.03%, 97.59% ± 0.09% and 95.19% ± 0.47%, respectively. As a sample pretreatment procedure, this method was coupled to high-performance liquid chromatography (HPLC) with good separation, sensitivity and linearity and was applied to determine the flavone content in different aerial parts of *S. baicalensis* and in dried blood spot samples.

## 1. Introduction

Flavonoids are common bioactive constituents in traditional medicine and dietary supplements, e.g., licorice and ginkgo. The determination of flavones and their metabolites in human and mammalian blood is important to understand their pharmacological characteristics. Baicalin, baicalein, wogonoside, wogonin and scutellarin are the main constituents in the plants of the genus *Scutellaria*. All these compounds are effective antioxidants [1]. Baicalin, baicalein and wogonoside show antitumor [2,3] and anti-HIV [4] effects. Scutellarin shows more potential in anti-inflammatory [5] and liver protection [6] functions. Plants in the genus *Scutellaria*, also known as skullcaps, are widely used herbs in Eastern and Western traditional medicine [7,8]. The above- and underground parts of *Scutellaria* are used by herbalists. The aerial part of *S. lateriflora* was used by Native Americans and Europeans as a nerve tonic, sedative, and anticonvulsant [8] and was proven to be a potential anti-diabetic agent [9,10]. The aboveground part of *S. baicalensis* is not used in traditional Chinese medicine, however, it is used as a type of tea beverage by locals [11] and has been widely studied in China. The major constituents in the roots of skullcaps are typically baicalin, baicalein, wogonoside and wogonin, while in the aboveground part, the primary constituent is scutellarin. Antioxidant potential studies showed that there were no significant differences in the aerial parts of *S. baicalensis*, *S. lateriflora* and *S. racemosa* [7]. The isolation and determination of the bioactive constituents in different parts of skullcaps would be beneficial to better understand the diversity of their bioactivities and to promote their applications. However, chlorophyll-type impurities are typically a tremendous obstacle for the ingredient purification and analysis from the aerial parts of plants [12]. There are many procedures established for chlorophyll removal from the extract of plant samples, e.g., liquid extraction, precipitation under low temperature and solid phase extraction [13,14,15,16]. However, these processes are either time consuming or result in analyte loss.

Dried blood spot (DBS) has been a favorite species of clinic test [17,18]. As a method for blood sample collection and clinical test storage, DBS offers many advantages, e.g., simple transfer with low infection risk and requirement of a small blood volume. However, its application has been hampered by the sample pretreatment, which has generated considerable interest over the past decades [19]. Like the chlorophyll in the aerial parts of plants, heme is also a porphyrin complex contaminant in whole blood analysis. Heme-type background substances are typically avoided by using plasma or serum [20], however they are one of the major contaminants in DBS. A rapid and efficient method for flavone isolation from a whole blood sample would remarkably facilitate the pharmacological studies of flavones [17,18], e.g., by reducing the number of operating steps, minimizing sample consumption, and simplifying the sample transformation and storage requirements. In our previous work [21], some of the flavones in *Radix Scutellariae* were proven to form complexes with the zinc cation at a high rate. These flavone-metal chelates are insoluble and can be precipitated from the solution, while other background substances, e.g., chlorophylls, lipids and waxes, which cannot form insoluble metal complexes, can be conveniently and efficiently separated from the flavones.

In this study, the conditions for separating scutellarin, baicalin, baicalein, wogonoside and wogonin from biological samples using zinc acetate were optimized. The theory of this method was described in our previous work [21]. The procedure to deposit flavones and remove background substances on dried blood spot (DBS) samples was also optimized. This technique was successfully coupled to high-performance liquid chromatography (HPLC) to determine the flavone content in different aerial parts of *S. baicalensis* and in DBS samples.

## 2. Results

### 2.1. Optimizing Conditions for Flavone Isolation Using Zinc Salt

#### 2.1.1. Recovery of the Isolation Procedure

Baicalin and baicalein has been precipitated from the ethanol extract of *Radix Scutellariae* with zinc acetate in neutral solution and shown good recoveries [21,22], while the recoveries of wogonoside and wogonin have not shown satisfactory results, according to our previous work [21]. Ammonium hydroxide has now been used to adjust the pH value of the reaction solution to obtain better recoveries of wogonoside and wogonin.

**Figure 1:**
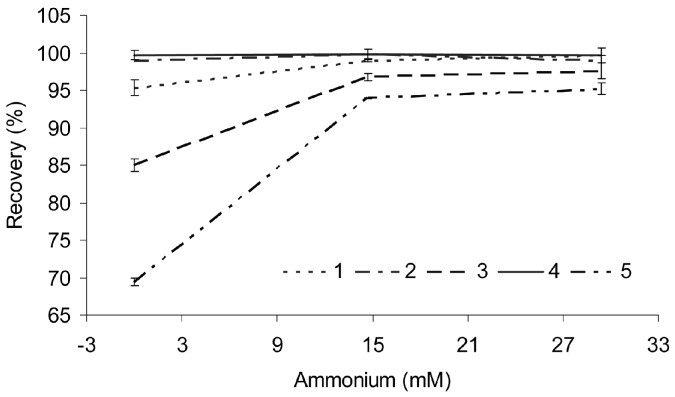
Effects of ammonium hydroxide on the recoveries of flavones (*n* = 3). Peaks: 1 = scutellarin; 2 = baicalin; 3 = wogonoside; 4 = baicalein; and 5 = wogonin.

Different amounts of ammonium hydroxide were added to the extract and mixed well prior to treating the sample with zinc acetate. The recoveries of scutellarin, baicalin, baicalein, wogonoside and wogonin are shown in Figure 1. The results indicate that these flavones are recovered very well under the conditions in which the contents of ammonium hydroxide in the reaction solution were more than 14.7 mM (0.025%). Under this pH condition, the mixture is stable, and the flavones can be stored for months at 4 °C [23]. The flavones can be obtained in a very good recovery at a very low concentration.

#### 2.1.2. Concentration Limit of the Separation

**Figure 2:**
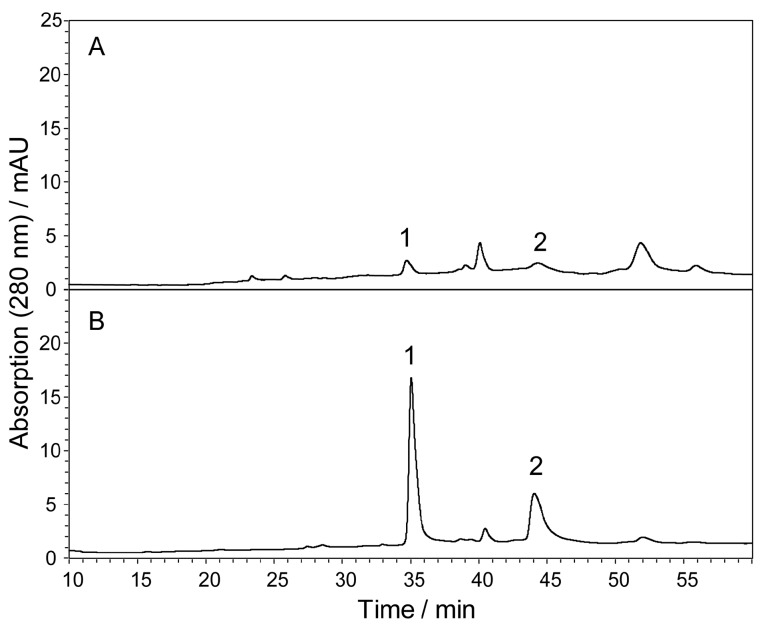
Representative chromatogram of the extract of the *S.* *baicalensis* root with a low concentration of baicalin (230 ng·mL^−1^) before (**A**) and after (**B**) zinc acetate pretreatment. The flavones were concentrated five times after treatment. The recoveries of the other compounds, e.g., baicalein, were not calculated because their signals in the untreated solution were too weak to be integrated. The chromatograms were recorded at 280 nm using the method described in our previous study [21]. The injection volume was 100 µL. 1 = baicalin; 2 = baicalein.

In Figure 2, the concentration of baicalin in the solution (Figure 2A) is approximately 230 ng·mL^−1^. Zinc acetate was added to this solution (1:1) in a test tube. The supernatant of the mixture was moved from the tube after centrifugation. The tube was washed with 200 µL formic acid (1%, *v*/*v*). This solution was then introduced into the instrument for HPLC analysis (Figure 2B). The recovery of baicalin was the same as that in Figure 1 (approximately 99%) under these conditions, although virtually no sediment was detected.

#### 2.1.3. Repeatability of the Separation

As shown in Figure 1, the recoveries of different compounds in different experiments were highly stable. The RSD (%) of the recoveries of the five analytes obtained in different extractions was used to evaluate the repeatability of the method. All of the RSDs were equal to or less than 1.5%. These results indicated that the repeatability of the extraction is good.

### 2.2. Validation of the HPLC Procedure

#### 2.2.1. Selectivity 

**Figure 3:**
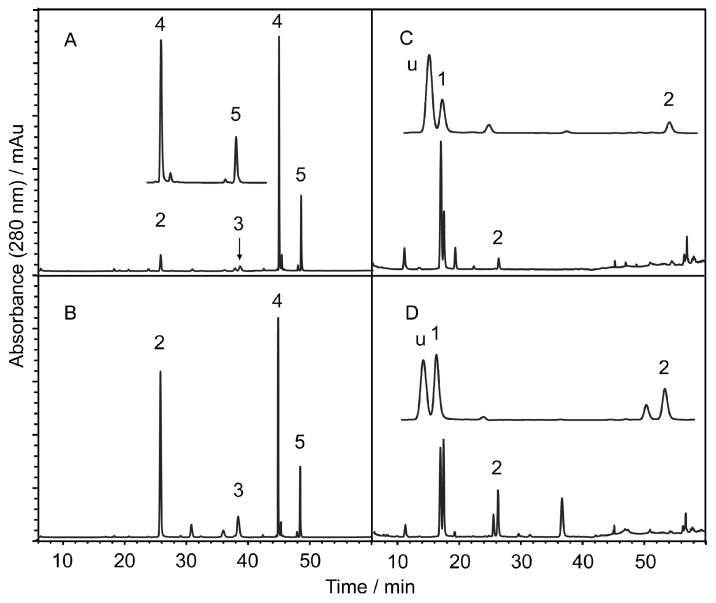
The representative chromatograms of different parts of *S. baicalensis*. (**A**) root bark; (**B**) taproot; (**C**) pod; (**D**) corolla; 1 = scutellarin; 2 = baicalin; 3 = wogonoside; 4 = baicalein; 5 = wogonin; u = unknown compound. All of the chromatograms are recorded at 280 nm.

The calculations of the capacity factor (k’), theoretical column plate number (N) and resolution factor (Rs) were the same as those used in the methods of our previous work [24]. Figure 3 shows the representative chromatograms of different parts of *S. baicalensis*, which are simplified after the removal of background substances.

**Figure 4:**
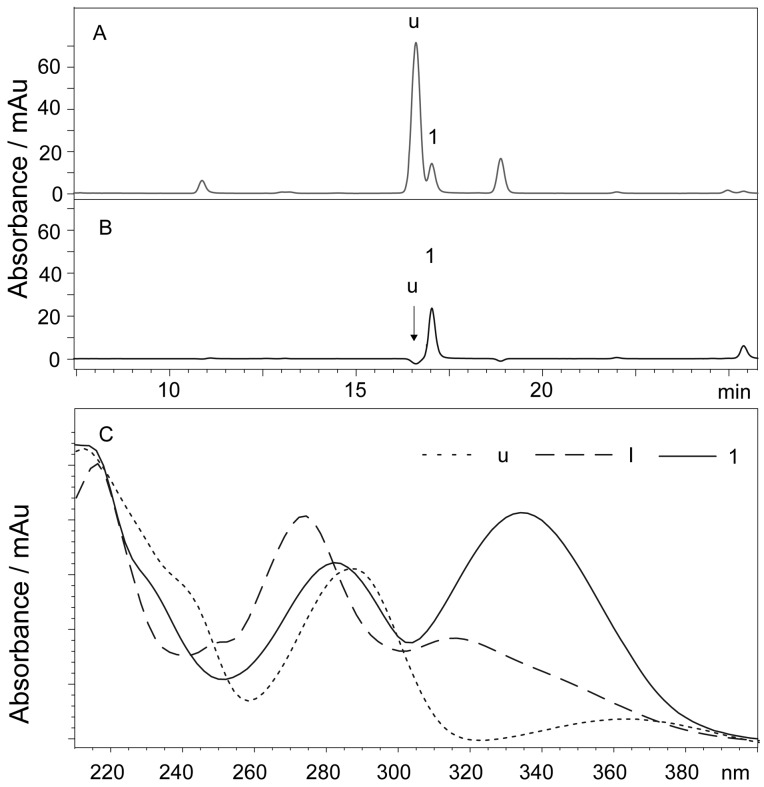
Chromatogram of the leaf of *S.* *baicalensis* recorded at 280 nm (**A**), 335 nm (**B**) and the UV spectra of compounds u, I and 1 (**C**). Compound 1 has a maximum absorption at 335 nm, whereas the absorption of compound u is very low. The chromatographic signal of compound u was not observed when the chromatogram was recorded at 335 nm. 1 = Scutellarin; u = unknown compound; I = 8-arabinosyl-6-glucosyl-5,7-dihydroxyflavone.

The k’, N and Rs of compounds 1 to 5 are presented in Table 1. There is a peak (16.8 min, *m*/*z* 463 [M − H]^−^) that is partially overlapped with compound 1 in the chromatograms of the stem, leaf, pod and corolla, and the Rs value of these two compounds was approximately 1.31 under the experimental conditions. Figure 4 shows the chromatograms of the leaf of *S. baicalensis* recorded at 280 nm (Figure 4A) and 335 nm (Figure 4B). The quantification of compound 1 did not experience interference by compound u when the signal was recorded at 335 nm because the absorptions of these two compounds are different at this wavelength (Figure 4C). There is no peak whose Rs value is less than 2.0 near the peaks of baicalin, wogonoside, baicalein and wogonin in the chromatograms obtained at 280 nm. Compared with the results in our previous study [21], the pentafluorophenyl column (PFP) shows a higher performance for flavonoid separation. The theoretical column plate numbers were more than 40,000 (m^−1^) for the compound, even though its k’ value was only 2.36 at a very low flow rate (0.5 mL·min^−1^).

The k’, N and Rs of compounds 1 to 5 are presented in Table 1. There is a peak (16.8 min, *m*/*z* 463 [M − H]^−^) that is partially overlapped with compound 1 in the chromatograms of the stem, leaf, pod and corolla, and the Rs value of these two compounds was approximately 1.31 under the experimental conditions. Figure 4 shows the chromatograms of the leaf of *S. baicalensis* recorded at 280 nm (Figure 4A) and 335 nm (Figure 4B). The quantification of compound 1 did not experience interference by compound u when the signal was recorded at 335 nm because the absorptions of these two compounds are different at this wavelength (Figure 4C). There is no peak whose Rs value is less than 2.0 near the peaks of baicalin, wogonoside, baicalein and wogonin in the chromatograms obtained at 280 nm. Compared with the results in our previous study [21], the pentafluorophenyl column (PFP) shows a higher performance for flavonoid separation. The theoretical column plate numbers were more than 40,000 (m^−1^) for the compound, even though its k’ value was only 2.36 at a very low flow rate (0.5 mL·min^−1^).

The peak purity analysis with respect to the detector response and the factor analysis for all of these compounds was evaluated using ESI-MS. The average ESI-MS spectra in the range of each peak of compounds 1 to 5 indicate that there was no impurity found in any of these peaks. The peak purity factors were automatically calculated using an off-line ChemStation Data Analysis program (Revision-B.01.03, Agilent, Santa Clara, CA, USA), and the values were within the threshold of 998. Compared with the previous study [25], the results of the purity of the detector response evaluation indicate that the selectivity of this method was suitable. Most importantly, this method is stable and specific for the determination of scutellarin, baicalin, wogonoside, baicalein and wogonin.

#### 2.2.2. Linearities, Limits of Detection and Limits of Quantification

**Figure 5:**
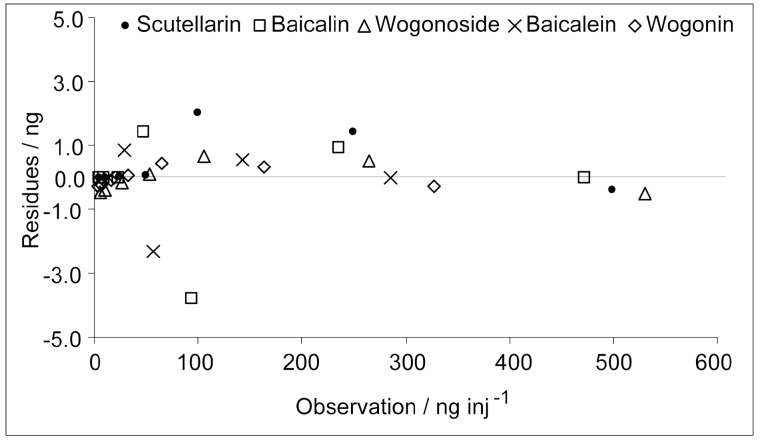
Residues of the calibration curves of the five analytes. The observation represents the amounts of the analytes introduced into the instrument for analysis. Residue is the difference between the observation and the fitted value, which is calculated from the average detector response. The maximum values of scutellarin, baicalin and wogonoside were approximately 500 ng·inj^−1^; those of baicalein and wogonin were approximately 300 ng·inj^−1^.

The five flavones showed good linearities of the detector response to the amount present. The parameters of the linear regression of all of these compounds are shown in Table 2. To avoid a large linearity parameter, each peak area was scaled down to 1%. The limits of detection (LOD) for the five compounds, based on the signal/noise ratio (*S*/*N*) was 3, which was obtained from a series of detector responses. The amounts (per injection) of these compounds that produced an *S*/*N* greater than 10 were accepted as the limits of quantification (LOQ). The LODs and LOQs of the five compounds are shown in Table 2. The relative standard deviations (RSD%) of the peak areas of the five compounds at the LOD concentration were not larger than 8.0%, and those of LOQ were not larger than 4.0%. Figure 5 shows the residual plot of the calibration curves. The absolute value of the residues were typically 0.02%–0.50% of the corresponding injection amounts. None of the values was greater than 5.0% of the observation. These observations indicated that the calibration of all five analytes was accurate.

### 2.3. Determination of Baicalin, Baicalein, Wogonoside and Wogonin in Different Parts of S. baicalensis

#### 2.3.1. Effects of Zinc Pretreatment on the Selectivity of the Quantification

**Figure 6:**
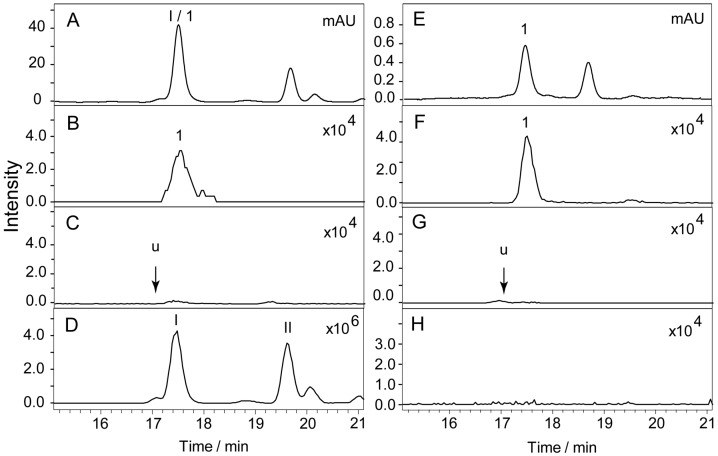
The UV chromatogram (335 nm (**A**)) and extracted ion chromatogram (EIC) at *m*/*z* 461 (**B**), *m*/*z* 463 (**C**) and *m*/*z* 547 (**D**) of the ethanol extract of the tap root of *S. baicalensis* and zinc acetate-pretreated extract (**E–H**); Compounds I and II removed by zinc acetate treatment (**H**); 1 = scutellarin; I = 8-arabinosyl-6-glucosyl-5,7-dihydroxyflavone; II = 6-arabinosyl-8-glucosyl-5,7-dihydroxyflavone; Compounds I and II were identified by their retention times according to the literature [21].

Figure 6 shows the UV chromatogram (335 nm Figure 6A), and the extracted ion chromatogram (EIC) at *m*/*z* 461 (Figure 6B), *m*/*z* 463 (Figure 6C) and *m*/*z* 547 (Figure 6D) of the ethanol extract of the *S. baicalensis* tap root. Scutellarin is typically present in very low quantities in the root of *S. baicalensis*, with a value of approximately 0.1% [26]. Typically, it was merged with 8-arabinosyl-6-glucosyl-5,7-dihydroxyflavone (I [21]), whose detector responses (UV and MS) are markedly higher than that of scutellarin, and they are difficult to separate. Many efforts were required to separate compound I from scutellarin to quantitatively determine scutellarin using an HPLC-UV procedure. In the chromatogram of the zinc acetate-pretreated sample (Figure 6E–H), the signal of compound I was not detected (Figure 6H). Compound I was depleted after the sample was treated with zinc acetate because it is a flavone C-glycoside, which does not form a complex with the zinc cation under these experimental conditions [21]. Compound u was not detected in the root of *S. baicalensis* (Figure 6C,G).

#### 2.3.2. Quantification of Scutellarin, Baicalin, Baicalein, Wogonoside and Wogonin

Table 3 shows the contents of scutellarin, baicalin, baicalein, wogonoside and wogonin in different parts of *S. baicalensis.* The bacailin content is approximately 14% in the tap root, which is consistent with the results in the literature [27,28]. The contents of scutellarin in different parts of *S. baicalensis* were also consistent with previously reported results [26]. Interestingly, the contents of the more hydrophilic scutellarin in the aerial parts were higher than that in the underground parts. The contents of baicalein and wogonin, which are less hydrophilic, are much higher than that of baicalin and wogonoside in the root bark. These aglycones might be the hydrolysates of baicalin and wogonoside in the bark (Figure 3A).

### 2.4. Determination of Scutellarin, Baicalin, Wogonoside in Artificial Dried Blood Spots

**Figure 7:**
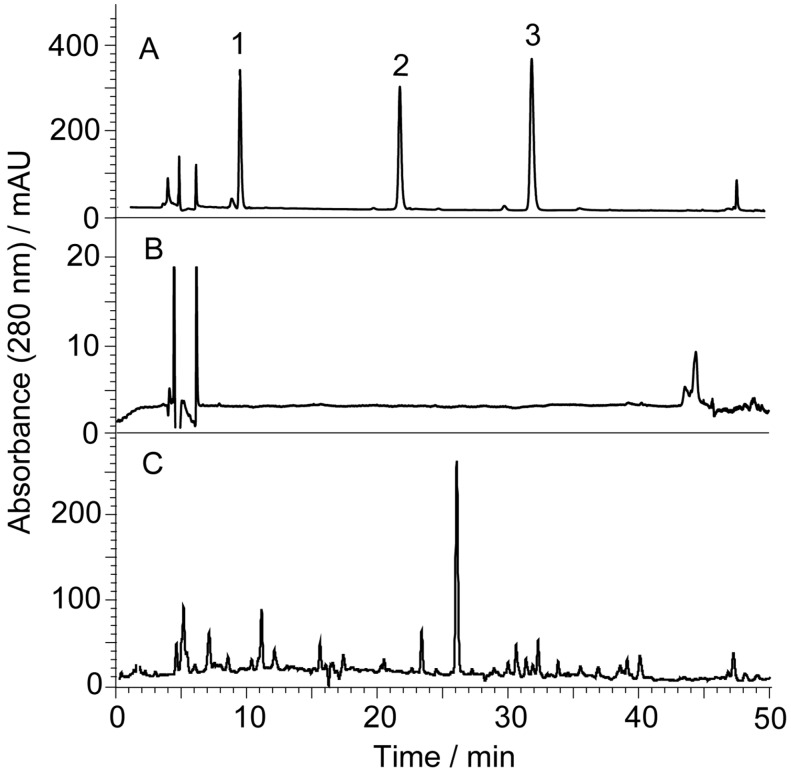
Representative chromatograms of zinc acetate-pretreated spiked artificial dried blood spots (**A**) and blank dried blood spots (**B**), and blank plasma treated using conventional method (**C**). All of the chromatograms were recorded at 280 nm. Background substances were successfully removed in the pretreatment procedure.

To ensure the amount of the spiked analytes on the DBS, a solution of reference materials was directly added onto the filter paper when the DBS was prepared. Figure 7 shows the chromatograms of flavones separated from the spiked DBS using this method. The recoveries of scutellarin, baicalin, and wogonoside were 65.7% ± 1.5%, 83.2% ± 1.4% and 73.8% ± 1.6%, respectively. When baicalin, wogonoside and wogonin were loaded directly on the pre-column with the extract of blank DBS, the peak areas were almost the same as those of the pure reference materials. The results indicated there was not matrix effect on the detector responses of the flavones after the DBS was treated with our method. The chromatogram of the blank sample also indicated that the extract of the blood was very clean after the treatment.

## 3. Discussion

Chlorophyll in the aerial parts of plants is a serious contaminant for isolation and analysis of plant components due to its high solubility [12,16]. This kind of background substance does not form insoluble complexes with zinc cation, so flavones with 3-hydroxyl, 5-hydroxyl or *ortho*-hydroxyl groups can be derivatized and separated from chlorophyll conveniently by simple centrifugation. Other background contaminants, e.g., proteins and lipid, can also be removed in the same time.

The current procedure involves many sample pretreatment steps to clean up the samples prior to DBS analysis. In addition to the complexity of the background substances, the amount of a sample is usually very small [19]. It is necessary to introduce all the samples into the HPLC to obtain good signal of the analytes. In this study, flavones were deposited on filter paper in the form of insoluble zinc complexes. The background substances, which did not form insoluble complexes with zinc, were washed using water and methanol. The blank plasma of mice, which was treated using a conventional method, was also analyzed for comparison (Figure 7C). Compared with the conventional method, the interference signals were dramatically removed in the chromatograms of DBS treated using this procedure (Figure 7A,B). These analyses indicated that the background removal using this procedure is highly effective. The chromatogram of the artificial DBS (Figure 7A) showed that most of these compounds were intact and could be applied for further analyses. However, the volume of zinc acetate solution should be as low as possible because the lower the volume of the solution, the less analyte loss. The analyte amounts in DBS are typically very small, which will generate signals at a low signal-to-noise ratio if an insufficient amount of sample is injected into the instrument for analysis. We solved this problem by loading all of the analyte onto a reversed phase pre-column then connecting the pre-column to an analytical column for HPLC analysis.

## 4. Materials and Methods

### 4.1. Reagents and Materials

*Scutellaria baicalensis* Georgi was collected in late July from the herb garden of the Institute of Wild Animals and Plants, Chinese Academy of Agricultural Science (Jilin, China). The sepal, petal, pod, stem, leaf, hierbaculum, tap root, root bark and lateral root were separated when harvested. Reference materials, baicalin, baicalein, wogonoside and wogonin, were purchased from the Institute for Food and Cosmetics Control (Beijing, China). Scutellarin was purchased from Aladdin-Reagent Co. Ltd. (Shanghai, China). HPLC-grade acetonitrile was purchased from Fisher (Fair Lawn, NJ, USA). The purities of all of the reference materials were ≥98%. The other chemicals and regents used were the same as those in our previous study [21]. Unless otherwise stated, all of the chemicals were analytical grade.

### 4.2. Isolation of Flavones Using Zinc Acetate

The pH value of the flavone solution (400 µL) was adjusted to approximately 8–9 by adding an ammonium hydroxide solution (147.06 mM). Then, the solution was mixed with a zinc acetate solution (400 µL, 54.50 mM). The solution was vortexed for 2 min and then allowed to stand for approximately 8 min. The mixture was centrifuged for 5 min at 4000 rotations per minute (rpm) with a medicinal centrifuge. Then, the pellet was collected and was dissolved in 400 µL of 1.0% acetic acid (*v*/*v*, containing 30% methanol). The solution was injected into an HPLC system for analysis.

### 4.3. Isolation of Flavones from Different Parts of S. baicalensis Georgi

#### 4.3.1. Extraction

The tap root of *S. baicalensis* was dried in a hot air oven at 40 °C for 4 h. The other materials were dried in a room with good ventilation under ambient conditions. All of the dehydrated materials were pulverized. An aliquot of approximately 100 mg powder (≤0.5 mm) was extracted with 50 mL ethanol (50%, *v*/*v*) in an ultrasonic extractor for 30 min and then stored overnight at ambient temperature. Approximately 5 mL of the suspension was removed after vigorous shaking and was centrifuged at 4000 rpm for 10 min. The supernatant was removed for use.

#### 4.3.2. Isolating Flavones Using Zinc Acetate

The supernatant (400 µL) was mixed with ammonium hydroxide and zinc acetate solutions (54.50 mM). The final concentration of ammonium hydroxide was 14.7 mM. The total volume of the mixture was adjusted to 800 µL with 30% ethanol (*v*/*v*). The pellet was collected using the same protocol as previously stated. The pellet was dissolved in 400 µL of 1.0% acetic acid (*v*/*v*, containing 30% methanol). The solution was injected into an HPLC system for analysis.

### 4.4. Isolation of Flavones from Dried Blood Spots

#### 4.4.1. Preparation of Artificial Dried Blood Spots

In order to ensure that all the spiked reference materials remained on the dried blood spot, the solutions of reference material and the whole blood were added onto the filter paper directly. Solutions of baicalin, wogonoside and wogonin (5 µL, 5 mg·mL^−1^) was successively dropped onto a small piece of quantitative filter paper (φ 6 mm, Xinhua Paper Industry Co. Ltd., Hangzhou, China) and dried under ambient temperature. The blood was collected from a male SD rat by extirpation of the eyeballs after the animal was anaesthetized using ethyl ether. Then, the blood (approximately 10 µL) was dropped onto the filter paper until the blood was completely absorbed. The filter paper was dried under ambient temperature for use. For the plasma pretreatment, 200 µL plasma was diluted with 600 µL cold methanol. Then, the mixture was centrifuged at 10,000 rpm. The supernatant was collected and dried under a nitrogen flow at ambient temperature. The residue was dissolved in 100 µL methanol. The solution (5 µL) was injected into the HPLC for analysis. The experimental protocol was approved by the Animal Ethics Committee of the Beijing Institute of Technology, in accordance with the Principles of Laboratory Animal Care and Use in Res. (Ministry of Health, Beijing, China).

#### 4.4.2. Isolation of Baicalin, Wogonoside and Wogonin from Dried Blood Spots 

The filter membrane of a superfiltration tube was removed. The artificial dried blood spot was sandwiched between two filter papers and was fitted into the filter insert to replace the filter membrane. The filter papers were immersed using 3 µL ammonium hydroxide solution and then 15 µL zinc acetate (54.50 mM, containing 30% methanol) for 10 min. The hydrophilic background substances, e.g., proteins, animo acids, etc. were then removed by washing the papers with 100 µL ammonium hydroxide solution (14.7 mM, containing 30% methanol) and centrifuging the tube for 3 min at 4000 rpm. The hydrophobic background substances were removed with 100 µL of 70% methanol solution (containing 14.7 mM ammonium hydroxide) and centrifugation of the tube for 3 min at 4000 rpm. For flavones recovery, 30 µL of 1.0% acetic acid (containing 30% methanol) was added into the tube, and the tube was centrifuged for 3 min at 4000 rpm; this step was repeated for three times; the solution was then collected. All of the acidic solutions were combined. The solution was manually loaded into an HPLC pre-column (C18) using a manual sampling valve (7725i, Rheodyne, IDEX Health & Science, Oak Harbor, WA, USA). The pre-column was then connected to the analytical column for separation and detection. 

The effect of the blood matrix was tested by loading 5 µL of mixed reference material solution (baicalin, wogonoside and wogonin, 1:1:1) onto the pre-column that has been loaded the extract of the blank blood through the auto sampler for HPLC analysis. The peak areas of baicalin, wogonoside and wogonin were respectively compared to the corresponding peak areas.

### 4.5. Chromatography and Mass Spectrometry

An Agilent 1100 series LC MSD Trap system (Agilent) equipped with a quaternary-pump, an auto sampler, a diode array detector (DAD) and an SL trap mass spectrometer was employed for the analysis. The separation was achieved on a pentafluorophenyl column (PFP, 5 mm, 4.6 × 250 mm, Discovery HS F5, Supelco Inc. Bellefonte, PA, USA) using a gradient mobile phase. Phase A was ammonium formate buffer containing 0.5% (*w*/*v*) ammonium formate and 0.5% (*v*/*v*) formic acid (pH 4.4–4.5). Phase B was pure acetonitrile. The gradient program was as follows: 20% B was maintained for the first 5 min, then a linear gradient of 20%–25% B was used for 5–20 min, then 25% B was maintained for 20–25 min, 25%–50% B for 35–40 min, 50%–75% B for 40–55 min, 75%–95% B for 55–60 min, and this composition was maintained from 60 to 65 min, then returned to the initial condition in 5 min. The flow rate of the mobile phase was 0.5 mL·min^−1^ for the first 35 min, then the flow rate increased linearly to 0.8 mL·min^−1^ for 35–40 min, and this flow rate was maintained until the end of the run. The sample solution (2 µL) was injected into the HPLC for analysis. Ultraviolet (UV) spectra were recorded from 220 nm to 400 nm, and the chromatogram from the UV detector was recorded at 280 nm and 335 nm, respectively. The MS analysis to confirm the signals was performed under the same conditions as those used in our former experiment [21]. The column was regenerated every five runs by injecting 100 µL of 1% formic acid solution into the column and washing with the same program as that used in the sample analysis.

## 5. Conclusions

Complexation was used to rapidly and effectively separate flavones from the aerial and the underground parts of *S. baicalensis*, as well as from spiked dried blood spots. The recoveries of the flavones from the plant extract were greater than 95%, and the recoveries of the flavones from the DBS were greater than 65%. The background substances were removed efficiently. This method provides a useful method for flavone separation from biological samples. It is also rapid and precise for flavone determination in biological samples, especially in botanical aerial parts and mammalian blood.

## Figures and Tables

**Table 1:** Chromatographic parameters of compounds **1–5** (*n* = 3).

No	k’		N (m^−1^)		Rs *	[M − H] ^−^
	Average	RSD%	Average	RSD%		
**1**	2.36	1.08	40,718	2.02	1.31 ± 0.02	463
**2**	4.00	0.91	89,299	8.44	2.08 ± 0.00	445
**3**	6.50	1.49	54,385	8.51	3.79 ± 0.03	429
**4**	7.65	0.15	1,403,517	0.26	2.77 ± 0.12	269
**5**	8.33	0.10	1,290,340	5.08	2.87 ± 0.13	373

* R, resolutions of the analyte and the nearest peak in the chromatograms; k’, capacity factor; N, column plate number; RSD, relative standard derivation; **1** = scutellarin; **2** = baicalin; **3** = wogonoside; **4** = baicalein; and **5** = wogonin.

**Table 2:** Linearity, limits of detection and limits of quantification of the HPLC procedure (*n* = 3).

Compound	a	b	R^2^	LOD (ng·inj^−1^)	LOQ (ng·inj^−1^)	Range (ng·inj^−1^)
Scutellarin	0.0500 ± 0.0000	−0.5452 ± 0.2750	0.9999	2.50	4.99	4.99–499.00
Baicalin	0.0727 ± 0.0000	−0.8635 ± 0.0406	0.9999	2.36	4.72	4.72–472.00
Wogonoside	0.1096 ± 0.0001	0.0663 ± 0.0022	1.0000	1.33	5.30	5.30–530.00
Baicalein	0.1201 ± 0.0000	−0.8634 ± 0.0884	0.9999	1.55	3.10	3.10–310.00
Wogonin	0.1775 ± 0.0001	0.0668 ± 0.0192	1.0000	1.65	3.30	3.30–330.00

a and b are the parameters of the equation y = ax + b, where y is the detector response of the compounds (1% of the peak area), x is the amount of analyte (ng); R is correlation coefficient; LOD is the limit of detection; LOQ is the limit of quantification; and Range is the range of analyte for the calibrate curve.

**Table 3:** Contents of baicalin, baicalein, wogonoside and wogonin in different parts of *Scutellaria baicalensis* Georgi (%, *n* = 3).

Part	Scutellarin	Baicalin	Wogonoside	Baicalein	Wogonin
Pod	0.44 ± 0.05	0.11 ± 0.03	0.02 ± 0.00	0.01 ± 0.00	ND
Corolla	0.78 ± 0.09	0.27 ± 0.01	ND	ND	ND
Leaf	0.91 ± 0.11	0.01 ± 0.00	0.02 ± 0.00	0.01 ± 0.00	ND
Stem	0.37 ± 0.04	0.05 ± 0.01	0.02 ± 0.00	ND	ND
Hierbaculum	0.15 ± 0.02	5.73 ± 0.14	0.93 ± 0.00	0.65 ± 0.06	0.47 ± 0.15
Tap root	0.14 ± 0.02	14.38 ± 0.12	2.08 ± 0.00	1.79 ± 0.16	0.43 ± 0.15
Lateral root	0.16 ± 0.02	12.09 ± 0.30	1.73 ± 0.00	1.72 ± 0.15	0.42 ± 0.15
Root bark	ND	1.04 ± 0.03	0.38 ± 0.00	3.71 ± 0.32	1.33 ± 0.13

ND: Not detected.
